# Stability-based validation of dietary patterns obtained by cluster analysis

**DOI:** 10.1186/s12937-017-0226-9

**Published:** 2017-01-14

**Authors:** Nicolas Sauvageot, Anna Schritz, Sonia Leite, Ala’a Alkerwi, Saverio Stranges, Faiez Zannad, Sylvie Streel, Axelle Hoge, Anne-Françoise Donneau, Adelin Albert, Michèle Guillaume

**Affiliations:** 1 Luxembourg Institute of Health (LIH), CCMS (Competence center in methodology and statistics), 1A rue Thomas Edison, L-1445 Strassen, Luxembourg; 2 Ministry of Health, Luxembourg, Service épidémiologie & statistique, Allée Marconi, Villa Louvigny, L-2120 Luxembourg city, Luxembourg; 3 Département des maladies cardiovasculaires, Hypertension Unit, Centre Hospitalier Universitaire, 5, Rue du Morvan, 54500 Vandœuvre-lès-Nancy, France; 4 Ecole de Santé Publique, Université de Liège, 7, Place du 20 Août, 4000 Liège, Belgium

**Keywords:** Dietary patterns, Cluster analysis, Stability

## Abstract

**Background:**

Cluster analysis is a data-driven method used to create clusters of individuals sharing similar dietary habits. However, this method requires specific choices from the user which have an influence on the results. Therefore, there is a need of an objective methodology helping researchers in their decisions during cluster analysis. The objective of this study was to use such a methodology based on stability of clustering solutions to select the most appropriate clustering method and number of clusters for describing dietary patterns in the NESCAV study (*Nutrition, Environment and Cardiovascular Health*), a large population-based cross-sectional study in the Greater Region (*N* = 2298).

**Methods:**

Clustering solutions were obtained with K-means, K-medians and Ward’s method and a number of clusters varying from 2 to 6. Their stability was assessed with three indices: adjusted Rand index, Cramer’s V and misclassification rate.

**Results:**

The most stable solution was obtained with K-means method and a number of clusters equal to 3. The “Convenient” cluster characterized by the consumption of convenient foods was the most prevalent with 46% of the population having this dietary behaviour. In addition, a “Prudent” and a “Non-Prudent” patterns associated respectively with healthy and non-healthy dietary habits were adopted by 25% and 29% of the population. The “Convenient” and “Non-Prudent” clusters were associated with higher cardiovascular risk whereas the “Prudent” pattern was associated with a decreased cardiovascular risk. Associations with others factors showed that the choice of a specific dietary pattern is part of a wider lifestyle profile.

**Conclusion:**

This study is of interest for both researchers and public health professionals. From a methodological standpoint, we showed that using stability of clustering solutions could help researchers in their choices. From a public health perspective, this study showed the need of targeted health promotion campaigns describing the benefits of healthy dietary patterns.

**Electronic supplementary material:**

The online version of this article (doi:10.1186/s12937-017-0226-9) contains supplementary material, which is available to authorized users.

## Background

In recent years, the dietary patterns (DP) approach has been used extensively to describe overall eating patterns in populations. In the literature, the most famous methods for computing dietary patterns are cluster analysis (CA) and principal component analysis (PCA). However, both methods describe diet in quite different ways. In PCA, continuous factors are defined based on correlations between dietary intakes and each individual has a score for all derived factors [1]. However, an individual’s DP is difficult to interpret as it is described by a score on several factors [2]. On the other hand, cluster analysis separates individuals into mutually exclusive groups (clusters) based on similarities between their diets. Compared to factors, individual DP are easier to interpret since individuals are assigned to one cluster only.

One major challenge in using cluster analysis is that the obtained solution strongly depends upon the choices made by the investigator. Among them, the choice of the clustering method and the optimal number of clusters are particularly important [3]. Indeed, since different clustering methods make different assumptions about the structure of the data, the choice of the method should be done according to the group structure expected. However, researchers do not have any prior knowledge about the structure of the clusters and their number. As a result, it appears that researchers run different clustering methods with different number of clusters and tend to present the best interpretable solution [1, 2, 4]. Obviously, this solution may not be the best representative of dietary patterns in a population. As an alternative, some studies used indices measuring distances between clusters [3, 5–7]. However, since those indices assume a group structure, their use should be avoided when group structure is unknown [8].

Consequently, researchers need a method allowing objective selection of the most appropriate clustering method and number of clusters for describing their data. Lange et al. introduced an objective criterion to compare different clustering solutions and to choose the most appropriate [8]. This criterion measures the goodness of clustering solutions by assessing their stability. A stable clustering solution should be similar to solutions computed on other data sets drawn from the same source. The idea is that clustering solutions exhibiting higher stability are likely to be more appropriate for describing the data.

Therefore, the primary objective of this study was to test such objective procedure to select the optimal clustering method and the number of clusters describing dietary patterns, based on data from the interregional, cross-sectional population-based NESCaV study (Nutrition, Environment and Cardiovascular Health). For simplicity, we decided to limit its application to traditional clustering methods used in the field of dietary pattern analysis, namely K-means, K-medians and Ward’s minimum variance. Secondly, description of the selected clustering solution and relationships with nutrients intakes, socio-demographic, lifestyle and cardiovascular risk factors (CVRF) were presented. Finally, a comparison was also made with PCA factors.

## Methods

Details concerning the NESCAV study have been presented previously [9–11]. Briefly, it is the first cross-border cardiovascular health population-based study, based on a stratified random sample of 3133 subjects, aged 18–69 years, recruited from three neighboring regions, namely Grand-Duchy of Luxembourg, Wallonia in Belgium, and Lorraine in France, constituting an important segment of the Greater Region population. Periods of recruitment were 2007 to 2008 for Grand-Duchy of Luxembourg and 2010 to 2011 for Wallonia and Lorraine. Pregnant women, people living in institutions, subjects outside the age range 18–69 years and those deceased before recruitment were excluded [10]. Sample sizes were computed in order to be able to estimate prevalence of cardiovascular risk factor with a level of confidence of 95% and a precision of 1%.

A 134-food frequency questionnaire (FFQ) was used to assess dietary intakes. Description and validation of this questionnaire have been detailed elsewhere [12, 13]. To facilitate the analysis, the 134 food items were merged into 45 broader food groups according to their similarities (unpublished observations). Daily food intakes were computed as the product of daily frequency of consumption and the amount consumed. Considered cardiovascular risk factors (CVRF) were body mass index (BMI, kg/m^2^), waist to hip ratio (WHR), systolic blood pressure (SBP, mmHg), diastolic blood pressure (DBP, mmHg), fasting plasma glucose (FPG, mg/dl), glycated haemoglobin (HbA1c, %), low-density lipoprotein cholesterol (LDL, mg/dl), high-density lipoprotein cholesterol (HDL, mg/dl) and triglycerides (TG, mg/dl). Information on treatment for hypertension, diabetes and dyslipidaemia was also gathered. Collected lifestyle behaviours were smoking status and level of physical activity expressed as weekly energy expenditure in metabolic equivalent task minutes per week (METs min/week), based on self-reported data from the International Physical Activity Questionnaire (IPAQ) [14, 15]. Specific inclusion criteria for this particular study were also defined. Flowchart of participants who met inclusion criteria were described (see Additional file 1: Figure S1). First, 138 participants with non-reliable reporting in the FFQ and outlying values on nutrient intakes were excluded. Then, since relationships between dietary habits and CVRF may be biased by participants who had a serious cardiovascular event (*n* = 327) and/or who are under diet (*n* = 312), those individuals were excluded. In addition, participants who were not fasting at time of blood collection (*n* = 58) were also discarded. Thus, the final sample entailed 2298 individuals.

The protocol of the study was approved by the following institutional review boards: Comité National d’Ethique de Recherche (Grand-Duchy of Luxembourg), Comité de Protection des Personnes Est-III (Lorraine), Comité d’Ethique Hospitalo-Facultaire Universitaire de Liège (Wallonia) and Ethik-Kommission Ärztekammer des Saarlandes (Saarland). All participants provided written informed consent.

### Statistical analysis

#### Transformation of the data

Firstly, food groups and nutrient intake were adjusted for energy intake using the residuals methods of Willet and Stampfer [16]. Secondly, since extreme values may have a significant effect on clustering solutions, extreme intakes above six standard deviations were truncated [17]. Of the 103,410 available intakes, only 294 (0.28%) were truncated. Thirdly, since food intakes with large scales tend to have a larger effect on clustering solutions, food intakes were standardized by subtracting the minimum intake and then dividing by the range [3, 18].

#### Formalization of cluster analysis

Let X = (X_1_, …, X_n_) be the dataset of *n* = 2298 individuals to be clustered where X_i_ is a 45-dimensional vector containing the 45 standardized food group intakes of the i-th individual. A clustering algorithm A with a predefined number of cluster k constructs a solution Y of the data set X into k clusters (Y := A_k_(X)). This solution Y is represented by an n-dimensional vector of labels Y = (Y_1_, …, Y_n_) where Y_i_ = v if the i-th individual is assigned to cluster v (v ∈ {1, …, k}).

### The measure of stability

Cluster stability exploits the fact that when multiple datasets are sampled from the same distribution, the clustering algorithm is expected to behave in the same way and produce similar results. Based on this idea, Lange et al. introduced a stability measure computed on the comparison of solutions obtained on different datasets drawn from the same source [8]. This stability measure was then compared across clustering methods and numbers of clusters to select the model associated with the most stable solution. Since this concept and its use in practice were previously described in detail [8], the method is only summarized below.

Briefly, considering a solution Y := A_k_(X), the method consists in assessing its stability by randomly splitting the data X into two independent half sets X_tr_ (training dataset) and X_te_ (test dataset), and comparing the solutions obtained for these halves (Y_tr_ := A_k_(X_tr_) and Y_te_ := A_k_(X_te_)). However, since dataset X_tr_ and X_te_ are disjoint, clustering solutions are not directly comparable. To make these solutions comparable, a solution transfer mechanism allows extension of the clustering solution Y_tr_ of the dataset X_tr_ to the dataset X_te_. Technically, the training dataset (X_tr_,Y_tr_) is used to construct a classifier ɸ which is then used to predict label of individuals from the test sample X_te_. Consequently, the two clustering solutions A_k_(X_tr_) and A_k_(X_te_) are made comparable by comparing ɸ (X_te_) and A_k_(X_te_). The stability measure between the two solutions is then computed as the empirical misclassification rate [8]. Lower misclassification rates indicate higher stability.

In order to reduce the effect of random splitting, the algorithm was repeated 20 times and the estimates of stability for a given solution were computed as the average of the 20 corresponding estimates. The highest estimate of stability indicates the optimal clustering method and number of clusters. Clustering methods considered were the Ward’s minimum variance, K-means and K-medians and number of clusters k varying from 2 to 6. Since K-means and K-medians may return a local optimum, algorithms were always run 1000 times with different random starting seeds, and the solution that had the minimum total within-cluster sum of squares distances was selected. Concerning the choice of the classifier ɸ , since we want to measure the stability of clustering solutions, the influence of the classifier should be minimized. For this purpose, Lange suggested choosing a classifier using the same clustering method’s grouping principle [8]. Therefore, K-nearest-means classifier was used when K-means and Ward’s methods were assessed whereas the K-nearest-medians classifier was used for the K-medians algorithm. Moreover, as a sensitivity analysis for assessing the impact of the stability indices used, others measures, namely Cramer’s V and Adjusted Rand index (ARI) were also computed. Contrary to the misclassification rate, higher values on Cramer’s V and ARI indicate higher stability.

### Description of dietary patterns

According to the stability indices values, the optimal clustering method for describing dietary patterns in our dataset was K-means with a number of clusters equal to 3. Clusters were described with mean of daily food intakes relative to corresponding overall mean intake. Cluster names were assigned based on food groups with high consumption. Clusters were also presented according to nutrient intake, socio-demographic and lifestyle factors. Continuous variables were presented as mean ± standard deviation (SD). Since most of the variables describing food and nutrient intake were not normally distributed, differences across clusters were evaluated using Kruskal-Wallis test. Categorical variables were presented as percentages (%) and differences were tested by the Chi-square test. A multinomial logistic regression was run to assess the relationships between clusters (dependent variables) and all socio-demographics and lifestyle characteristics as independent variables. Finally, separate multivariable-adjusted regression models for each CVRF (dependent variables) were also used to assess relationships with clusters (independent variables). Models were adjusted for gender, age, educational level, smoking status and the level of physical activity and medication use for the corresponding CVRF. Interaction between DP and gender were tested and if significant, results were stratified by gender. In order to take into account the sampling design of the study, individuals were weighted by the reciprocal of the probability of selection. All analyses were conducted with SAS version 9.4 (SAS Institute Inc., Cary, NC, USA). Ward’s method was performed with the procedure PROC CLUSTER and K-means and K-medians with the procedure PROC FASTCLUS. *P*-values < 0.05 were considered as significant.

### Comparison with PCA-DP

Continuous dietary patterns were also computed with PCA method. PCA-DP scores were calculated as a sum of the food intake variables weighted by the loadings generated by the method. Food groups with absolute loadings values superior to 0.2 were considered as contributing highly to the pattern [2]. According to the elbow method, three dietary patterns were selected. Both methods PCA and cluster analysis were compared by comparing means of PCA-DP across clusters with the Kruskal-Wallis test.

## Results

### Choice of clustering method and number of clusters

**Fig. 1:**
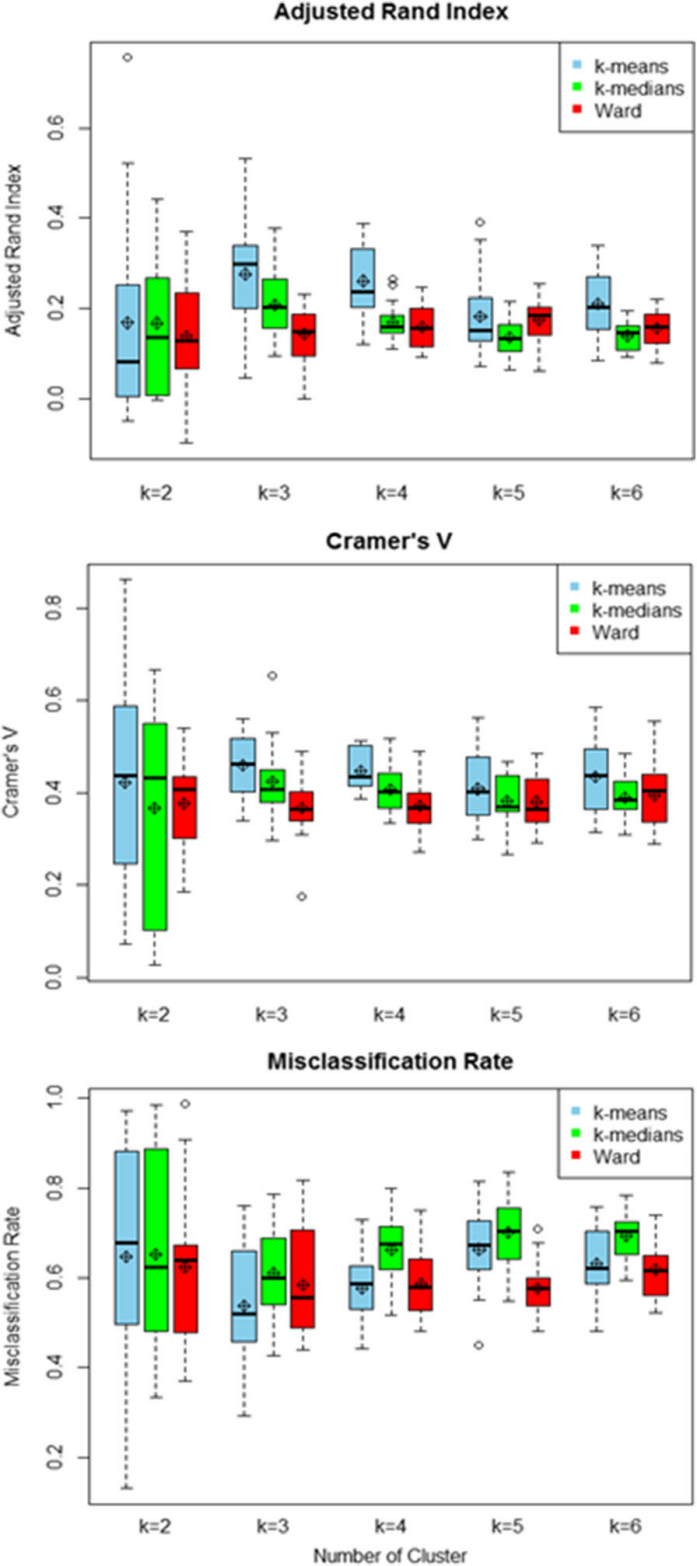
Distribution of stability indices across clustering methods and number of clusters

Figure 1 presents the distribution of the three stability indices across clustering methods and number of clusters. Distributions were described with box-plots and average values computed on 20 repetitions of the algorithm. Regardless of stability indices and number of clusters, more stable solutions were obtained with K-means. In addition, the most stable solution was obtained with 3 clusters. Therefore, dietary patterns were computed with K-means algorithm and a predefined number of clusters equal to three.

### Dietary patterns

The description of each cluster is given in Table 1. Clusters were described with mean of daily food intakes relative to corresponding overall mean intake. The cluster labelled “**Prudent**” was characterized by high intakes of brown bread, fruits, oleaginous fruits, dried fruits, soups, vegetables, pulses, preserved vegetables, offal, fish, smoked and canned fish, shellfish and mussels, dairy products, soya products, olive oil, oil-rich in omega 3 or 6, water and tea. In contrast, individuals in this cluster had low intakes of white bread, pastries, rice and pasta, fried foods, lean and fatty meat, processed smoked meat, processed meat, ready meals, minarine and margarine, fresh cream and dressing, sugar and sweets, salty biscuits, soft drinks, diet soft drinks, beer and aperitifs and spirits. Concerning the “**Non-Prudent**” cluster, individuals in this cluster consumed less cereals, rice/pasta, fruits, oleaginous fruits, dried fruits, vegetables, pulses, preserved vegetables, fish, smoked and canned fish, dairy products, soya products, olive oil and oil-rich in omega 3 or 6, light fresh cream and dressings, sugar and sweets, water, fruit or vegetable juice and tea. In contrast, the “Non-Prudent” cluster had high intakes of white bread, potatoes, fried foods, lean and fatty meat, offal, processed meat, shellfish and mussels, minarine and margarine, fresh cream and dressings, coffee, diet soft drinks, beer and wine. Finally, the “**Convenient**” cluster was characterized by consumption of convenient fast foods that require little preparation like cereals, pastries, rice and pasta, preserved vegetables, smoked and canned fish, ready meals, high-fat dairy products, soya products, fresh cream and dressings, sugar and sweets, salty biscuits, fruit or vegetable juice, soft drinks and aperitifs and spirits. In contrast, individuals in this cluster had low consumption of brown bread, potatoes, oleaginous fruits, soups, vegetables, pulses, offal, fish, shellfish and mussels, oil-rich in omega 3, coffee and wine.

**Table 1:** Description of clusters according to daily food intakes relative to corresponding overall mean intake (Mean (SD))

Food groups	Clusters			*p*-value
	Convenient (*n* = 1005;46%)	Prudent (*n* = 718;29%)	Non-Prudent (*n* = 575;25%)	
White bread	**−2.9 (27.6)** ^**ab**^	1.4 (41.6)^a^	**3.4 (36.4)** ^**b**^	0.003
Brown bread	**−9.6 (50.4)** ^**a**^	6.4 (69.4)^b^	**8.7 (76.1)** ^**ab**^	0.003
Cereals	**19.5 (104.6)** ^**a**^	−0.2 (95.9)^b^	**−33.9 (43)** ^**c**^	<0.0001
Pastries	**5.6 (29.8)** ^**a**^	**−6.1 (27.2)** ^**b**^	−2.2 (27.8)^c^	<0.0001
Potatoes	**−12.2 (39.3)** ^**a**^	0.6 (54.2)^b^	**20.6 (55.8)** ^**c**^	<0.0001
Rice pasta	**7.6 (47.5)** ^**a**^	**−5.1 (45.8)** ^**b**^	**−6.9 (38.4)** ^**b**^	<0.0001
Fried foods	2.2 (27.7)^a^	**−10.3 (21.2)** ^**b**^	**9.1 (38.7)** ^**c**^	<0.0001
Fruits	−10.6 (30.2)^a^	**26.2 (63.4)** ^**b**^	**−14.2 (29.5)** ^**c**^	<0.0001
Oleaginous fruits	**−6.2 (40.6)** ^**a**^	**13.9 (71.3)** ^**b**^	**−6.3 (45)** ^**a**^	<0.0001
Dried fruits	−27.7 (69.6)^a^	**63.4 (235.2)** ^**b**^	**−30.7 (73.8)** ^**c**^	<0.0001
Soups	**−16.1 (40.2)** ^**a**^	**23.2 (71.4)** ^**b**^	−0.8 (53.9)^c^	<0.0001
Vegetables	**−15.1 (30.3)** ^**a**^	**30.9 (59.8)** ^**b**^	**−12.1 (36.3)** ^**a**^	<0.0001
Pulses	**−7.6 (37.7)** ^**a**^	**13.2 (56.3)** ^**b**^	**−3.3 (56.5)** ^**a**^	<0.0001
Preserved vegetables	**−3.4 (67)** ^**a**^	**12.1 (99.6)** ^**a**^	**−9.2 (56.8)** ^**b**^	0.017
Lean meat	−0.9 (28.7)^a^	**−6 (30.6)** ^**b**^	**8.9 (31.6)** ^**c**^	<0.0001
Fatty meat	4.4 (34.5)^a^	**−16.1 (30.9)** ^**b**^	**12.3 (44.9)** ^**c**^	<0.0001
Offals	**−14.7 (50.4)** ^**a**^	**16.3 (99.9)** ^**b**^	**5.4 (96.6)** ^**a**^	0.006
Processed smoked meat	−3.6 (37.7)^a^	**−7.1 (43.3)** ^**b**^	**15.2 (57.6)** ^**c**^	<0.0001
Processed meat	2.5 (37.1)^a^	**−12.7 (30.6)** ^**b**^	**11.4 (49.3)** ^**c**^	<0.0001
Fish	**−11.1 (35.3)** ^**a**^	**21.4 (56.1)** ^**b**^	**−7.4 (40.7)** ^**a**^	<0.0001
Smoked and canned fish	**−2.9 (56.1)** ^**a**^	**11.4 (83.4)** ^**a**^	**−9.1 (51.9)** ^**b**^	<0.0001
Shellfish and mussels	**−8.4 (56)** ^**a**^	**6.4 (71.4)** ^**b**^	**6.8 (64.6)** ^**b**^	<0.0001
Eggs	−0.7 (42.5)^a^	−0.4 (45.6)^a^	1.7 (45.2)^a^	0.559
Ready meal	**9.6 (31.9)** ^**a**^	**−10.2 (24.2)** ^**b**^	−4.1 (25.5)^c^	<0.0001
High-fat dairy products	**2.3 (34.4)** ^**a**^	**2.1 (38.4)** ^**a**^	**−6.7 (32.7)** ^**b**^	<0.0001
Low-fat dairy products	−4.92 (62.8)^a^	**18.2 (84.9)** ^**b**^	**−14.2 (57.3)** ^**c**^	<0.0001
Soya products	**−24.7 (233.9)** ^**a**^	**83.1 (519)** ^**a**^	**−60.6 (45)** ^**b**^	<0.0001
Butter and low fat butter	−8.1 (40.3)^a^	2.2 (55.7)^a^	11.5 (70.3)^a^	0.080
Minarine and margarine	−11.8 (53.1)^a^	**−21.1 (54.4)** ^**b**^	**47.1 (117.8)** ^**c**^	<0.0001
Olive oil	−5.4 (49)^a^	**22.7 (76.4)** ^**b**^	**−18.8 (40.7)** ^**c**^	<0.0001
Oil rich in omega6	−12.2 (53.3)^a^	**27.7 (101)** ^**b**^	**−13.3 (54.7)** ^**c**^	<0.0001
Oil rich in omega3	**−6 (47.3)** ^**a**^	**8.3 (69.6)** ^**b**^	0.1 (56.5)^ab^	0.013
fresh creamand dressing	**7.2 (43.5)** ^**a**^	**−13 (32.9)** ^**b**^	**3.6 (37.4)** ^**a**^	<0.0001
Light fresh cream and dressing	**4.2 (77.1)** ^**a**^	**−4.4 (75.8)** ^**b**^	**−1.9 (69)** ^**b**^	<0.0001
Sugar and sweets	**5.7 (27.1)** ^**a**^	**−4.6 (24.5)** ^**b**^	**−4.1 (25.1)** ^**b**^	<0.0001
Salty biscuits	**9.4 (65.5)** ^**a**^	**−13.5 (38.3)** ^**b**^	0.4 (60.6)^c^	<0.0001
Water	−2.1 (58.5)^a^	**14.8 (65.2)** ^**b**^	**−14.8 (58.6)** ^**c**^	<0.0001
Coffee	**−23.1 (44.4)** ^**a**^	−10.5 (52.8)^b^	**53.6 (85.8)** ^**c**^	<0.0001
Fruit or vegetable juice	**13.9 (67.8)** ^**a**^	−4.7 (50.3)^b^	**−18.4 (38.5)** ^**c**^	<0.0001
Soft drinks	**15.6 (63.9)** ^**a**^	**−18.9 (23)** ^**b**^	−3.9 (48.5)^c^	<0.0001
Diet soft drinks	**7.6 (192.1)** ^**a**^	**−35.5 (108.2)** ^**b**^	**31 (249.9)** ^**a**^	<0.0001
Beer	−4.6 (35.8)^a^	**−12.6 (28.5)** ^**b**^	**23.8 (82.7)** ^**c**^	<0.0001
Wine	**−19.7 (54.6)** ^**a**^	−2.9 (80.6)^b^	**38.1 (134.9)** ^**c**^	<0.0001
Aperitifs and spirits	**−2.6 (41.5)** ^**a**^	**−3.5 (52.5)** ^**b**^	**8.9 (63.2)** ^**a**^	<0.0001
Tea	−53.6 (93.3)^a^	**135.2 (259.2)** ^**b**^	**−75.2 (68)** ^**c**^	<0.0001

Legend: ^abc^Means with same letters are not significantly different from each other; means that are in **bold** face are highest; means that are **underlined and bold** are lowest

The distribution of dietary patterns is also described in Table 1. The “Convenient” pattern was the most prevalent with 46% of the population assigned to this cluster. The remaining two clusters were smaller with 25% and 29% of the population belonging respectively to the “Non-Prudent” and “Prudent” cluster.

The description of dietary patterns according to nutrient intake is presented in Table 2. “Prudent” cluster was characterized by high intakes of all micronutrients, carbohydrates, total fiber and plant protein. In contrast, this cluster was associated with low intakes of alcohol, animal protein, added sugar and dietary cholesterol. Concerning fat profile, individuals in this cluster have higher MUFA: SFA (Ratio of monounsaturated fat to saturated fat) and PUFA: SFA (Ratio of polyunsaturated fat to saturated fat). On the opposite, “Non-Prudent” cluster had the highest intakes of alcohol, animal protein, and dietary cholesterol. It was also characterized by low intakes of carbohydrates, total fibre, added sugar, fat and all micronutrients and low MUFA: SFA and PUFA: SFA ratios. The “Convenient” pattern was associated with high intakes of carbohydrates, added sugar and fat and low intakes of alcohol, total fiber, plant and animal protein, β-carotene, vitamin E and iron.

**Table 2:** Description of clusters according to nutrient intakes relative to corresponding overall mean intake (Mean (SD))

Nutrient		Clusters			*p*-value
		Convenient	Prudent	Non-Prudent	
Macro-nutrients	Alcohol	**−25.3 (89.0)** ^**a**^	**−19.6 (111.2)** ^**a**^	**69.4 (188.1)** ^**b**^	<0.0001
	Carbohydrates	**2.2 (16.6)** ^**a**^	**1.1 (20.5)** ^**a**^	**−5.2 (18.3)** ^**b**^	<0.0001
	Total fiber	**−9.3 (21.5)** ^**a**^	**19.3 (32.5)** ^**b**^	**−7.9 (26.4)** ^**a**^	<0.0001
	Plant protein	**−5.5 (22.0)** ^**a**^	**8.3 (28.9)** ^**b**^	−0.6 (26.1)^c^	<0.0001
	Animal protein	**−1.9 (29.7)** ^**a**^	**−1.4 (35.1)** ^**a**^	**5.1 (31.3)** ^**b**^	<0.0001
	Added sugar	**27.6 (73.1)** ^**a**^	**−24.9 (52.1)** ^**b**^	**−17.0 (60.9)** ^**b**^	<0.0001
	Fat	**0.9 (17.0)** ^**a**^	**−0.2 (21.7)** ^**ab**^	**−1.3 (19.7)** ^**b**^	0.03
	Cholesterol	0.9 (26.2)^a^	**−4.8 (34.1)** ^**b**^	**4.4 (26.2)** ^**c**^	<0.0001
	Monounsaturated fat	**−0.4 (21.5)** ^**a**^	**3.4 (27.4)** ^**a**^	**−3.6 (20.5)** ^**b**^	0.0001
	Polyunsaturated fat	**−6.1 (31.7)** ^**a**^	**13.2 (51.3)** ^**b**^	**−5.9 (29.4)** ^**a**^	<0.0001
	Saturated fat	**0.9 (13.0)** ^**a**^	**−2.91 (15.9)** ^**b**^	**2.1 (12.8)** ^**a**^	<0.0001
	Ratio of monounsaturated fat to saturated fat	−2.1 (36.0)^a^	**8.2 (49.5)** ^**b**^	**−6.5 (34.1)** ^**c**^	<0.0001
	Ratio of polyunsaturated fat to saturated fat	**−8.3 (44.7)** ^**a**^	**19.4 (79.4)** ^**b**^	**−9.7 (39.9)** ^**a**^	<0.0001
Micro-nutrients	β-caroten	**−17.5 (56.1)** ^**a**^	**39.9 (106.5)** ^**b**^	**−19.2 (58.5)** ^**a**^	<0.0001
	Vitamin C	−8.3 (47.6)^a^	**31.4 (94.6)** ^**b**^	**−24.6 (43.8)** ^**c**^	<0.0001
	Vitamin E	**−4.7 (34.3)** ^**a**^	**8.6 (43.2)** ^**b**^	**−2.4 (38.3)** ^**a**^	<0.0001
	Iron	**−3.2 (16.5)** ^**a**^	**4.6 (19.3)** ^**b**^	−0.3 (18.2)^c^	<0.0001
	Vitamin D	−8.7 (93.8)^a^	**25.0 (101.4)** ^**b**^	**−15.9 (64.8)** ^**c**^	<0.0001
	Calcium	−2.5 (26.5)^a^	**13.3 (37.3)** ^**b**^	**−12.4 (28.0)** ^**c**^	<0.0001

^abc^Means with same letters are not significantly different from each other; means that are in **bold** face are **highest**; means that are **underlined and bold** are **lowest**

### Association of DP with sociodemographic and lifestyle characteristics

The associations of DP with sociodemographic and lifestyle characteristics are shown in Table 3. “Non-Prudent” and “Convenient” clusters were compared to the “Prudent” cluster which was considered as the reference. Older subjects were less likely to adopt a “Convenient” pattern (OR = 0.92 [0.91; 0.93]). Indeed, individuals in the “Convenient” cluster were much younger (36.9 years) than those in the “Prudent” (49.3 years) and “Non-Prudent” (48.9 years) cluster. Men were also more likely to adopt a “Convenient” (OR = 2.2 [1.6; 3.1]) or “Non-Prudent” (OR = 4.2 [2.9; 5.9]) patterns rather than a “Prudent” one. Likewise, individuals with less education were also more likely to adopt a “Non-Prudent” pattern. Concerning the region, compared to Lorraine, individuals living in Luxembourg were more likely to adopt a “Convenient” (OR = 1.7 [1.2; 2.4]) or a “Non-Prudent” (OR = 2.1 [1.3; 3.4]) pattern. The difference was even larger when comparing with individuals living Wallonia with a net preference for the “Non-Prudent” (OR = 7.1 [4.5; 11.4]) and “Convenient” (OR = 2.7 [1.8; 3.9]) pattern. In details, 41% of individuals living in Lorraine adopted a “Prudent” pattern whereas they were only 28.7% in Luxembourg and 19.1% in Wallonia. On the opposite, only 14% of individuals in Lorraine adopted a “Non-Prudent” pattern whereas they were 19.9% in Luxembourg and 36.7% in Wallonia. Concerning lifestyle factors, smokers were more likely to adopt a “Non-Prudent” (OR = 3 [1.9; 4.7]) pattern. Regarding physical activity, individual in the “Convenient” cluster were engaged in significantly less physical activity (OR = 0.993 [0.988; 0.998]).

**Table 3:** Associations of clusters with sociodemographic and lifestyle characteristics (Mean(SD); Percentage) and odds-ratios)

Sociodemographic and lifestyle characteristics		Cluster			*p*-value	Odds ratio	
		Convenient	Prudent	Non-Prudent		Convenient vs Prudent	Non-Prudent vs Prudent
Age (years)	**(** ***n*** **= 2298)**	**36.9 (0.3)** ^**a**^	**49.3 (0.5)** ^**b**^	48.9 (0.6)^c^	<0.0001	0.92 [0.91;0.93]	1.00 [0.98;1.01]
Energy expenditure per week (MET/100)	**(** ***n*** **= 2298)**	**29.2 (1.21)** ^**ac**^	**33.6 (1.8)** ^**b**^	32.4(1.6)^c^	0.0075	0.993 [0.988;0.998]	0.996 [0.99;1.002]
Gender (%)	**Men (** ***n*** **= 1158)**	52.2%	**35.9%**	**66.0%**	<0.0001	2.2 [1.6;3.1]	4.2 [2.9;5.9]
	**Women (** ***n*** **= 1140)**	47.9%	**64.1%**	**34.0%**		Reference	
Educational level (%)	**Primary (** ***n*** **= 331)**	**7.2%**	10.1%	**10.5%**	<0.0001	1.3 [0.9;2.1]	2.9 [1.4;3.8]
	**Secondary (** ***n*** **= 1127)**	46.4%	**42.8%**	**57.6%**		1.9 [1.4;2.7]	2.5 [1.7;3.7]
	**Tertiary (** ***n*** **= 818)**	**46.4%**	41.1%	**32.0%**		Reference	
Smokers (%)	**Smokers (** ***n*** **= 484)**	21.6%	**15.3%**	**36.9%**	<0.0001	1 [0.6;1.6]	3 [1.9;4.7]
	**Non smokers (n = 1814)**	78.4%	**84.7%**	**63.1%**		Reference	
Region (%)	**Luxembourg (** ***n*** **= 1071)**	**26.3%**	23.0%	**19.1%**	<0.0001	1.7 [1.2;2.4]	2.1 [1.3;3.4]
	**Wallonia (** ***n*** **= 750)**	38.3%	**26.0%**	**60.0%**		2.7 [1.8;3.9]	7.1 [4.5;11.4]
	**Lorraine (** ***n*** **= 477)**	35.5%	**50.9%**	**20.9%**		Reference	

Legend: ^abc^ Means with same letters are not significantly different from each other; figures that are in **bold** face are **highest**; figures that are **underlined and bold** are **lowest**

### Association of DP with CVRF

Multivariate-adjusted β-coefficients for CVRF according to DP are displayed in Table 4. Compared to the “Prudent” pattern, higher BMI was noticed in individuals who adopted the “Convenient” and the “Non-Prudent” pattern whereas higher WHR was only observed in men having adopted the “Non-Prudent” pattern. “Non-Prudent” and “Convenient” patterns also showed higher SBP and DBP values. Concerning diabetes, “Convenient” and especially “Non-Prudent” patterns were significantly associated with higher FPG but not HbA1c. Regarding cholesterol levels, “Non-Prudent” cluster was associated with higher LDL and HDL in men only. Further adjustment of treatment did not change the results.

**Table 4:** Association of clusters with cardiovascular risk factors (β-coefficients (Standard error) of cluster on CVRF)

		Cluster	
		Convenient	Non-Prudent
CVRF	Model	β (standard error)	
BMI	M1	0.49 (0.24)*	1.20 (0.26)**
WHR	M1 men	0.01 (0.01)	0.02 (0.01)*
	M1 women	0.01 (0.005)	−0.002 (0.01)
SBP	M1	2.58 (0.79)*	4.41 (0.87)**
	M2	2.10 (0.77)*	4.04 (0.85)**
DBP	M1	1.54 (0.55)*	3.15 (0.61)**
	M2	1.29 (0.55)*	2.95 (0.60)**
FPG	M1	1.77 (0.88)*	3.10 (0.97)*
	M2	1.46 (0.78)	2.30 (0.86)*
Hba1c	M1	0.01 (0.02)	0.03 (0.03)
	M2	0.01 (0.02)	0.01 (0.02)
HDL	M1 men	−0.78 (1.09)	3.57 (1.10)*
	M1 women	0.07 (1.17)	−1.59 (1.44)
	M2 men	−0.82 (1.09)	3.62 (1.10)*
	M2 women	−0.11 (1.17)	−1.75 (1.44)
LDL	M1 men	1.18 (2.70)	6.63 (2.72)*
	M1 women	−2.77 (2.22)	−4.59 (2.73)
	M2 men	1.02 (2.69)	6.81 (2.71)*
	M2 women	−2.64 (2.22)	−4.47 (2.73)
TG	M1	−1.77 (4.34)	2.91 (4.78)
	M2	−1.25 (4.34)	2.94 (4.77)

Prudent cluster is the reference category

M1 : adjusted on gender, age, educational level, smoking status, physical activity

M2 : M1 + treatment for the studied CVRF

*p < 0.05

**p < 0.0001

### Comparison of dietary patterns obtained with PCA and K-means

**Fig. 2:**
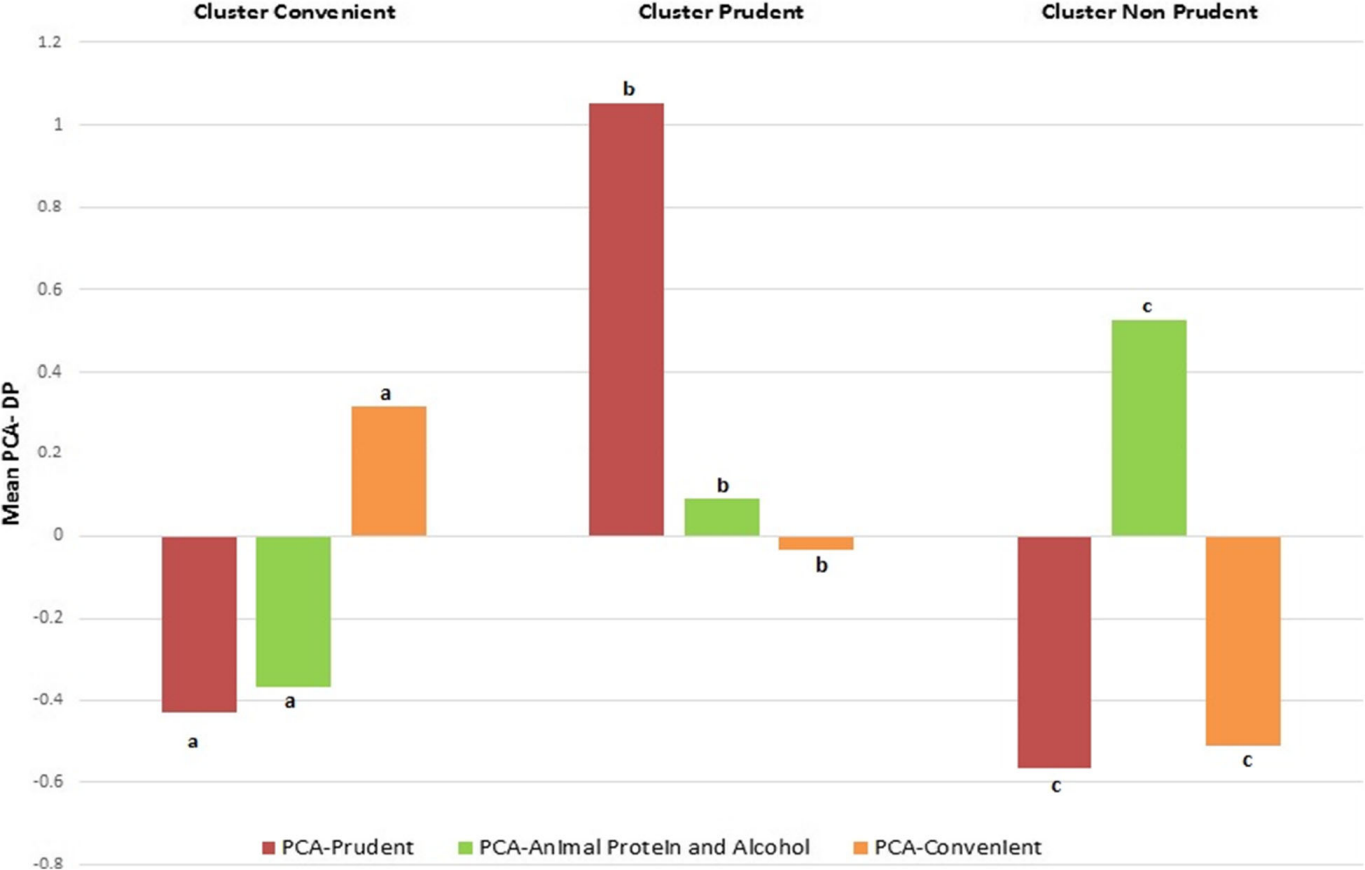
Mean PCA-DP across clusters. Legend: ^abc^test if differences in mean for a PCA-DP were significantly different across clusters. Means with same letters are not significantly different from each other

Continuous dietary patterns were computed using the PCA method. According to the scree-plot, three dietary patterns were selected. The percentage of variance explained and loadings of food groups on DP are presented in Table 5. The three PCA-patterns accounted for 7.1% (3.1%, 2.1% and 1.9% respectively) of the total variance in food intakes. The first pattern was labelled “Prudent” as it was characterized by high intakes of fruits, oleaginous and dried fruits, soups, vegetables, pulses, fish, low-fat dairy products, soya products, olive oil, oil-rich in omega 6, water and tea and low intakes of fried foods, lean and fatty meat, processed meat, ready meals, minarine and margarine, fresh cream and dressing, salty biscuits, soft drinks, diet soft drinks and beer. The second PCA-pattern was named “Animal protein and alcohol” since it was positively associated with vegetables, pulses, all kinds of meat and fish and alcohol beverages and negatively associated with sugar and sweets, high-fat dairy products, pastries and cereals. The third pattern was labelled “Convenient” since this pattern was positively correlated with convenient foods that require little preparation like brown bread, cereals, rice, pasta, smoked and canned fish, shellfish and mussels, ready meals, low-fat dairy products, soya products, fresh cream and dressings, salty biscuits, fruit or vegetable juice. Moreover, it was also negatively correlated with white bread, potatoes and butter. Comparison of dietary patterns obtained through PCA and K-means are shown in Fig. 2. The three clusters were similar to the three continuous dietary patterns obtained through PCA. Indeed, the PCA-Prudent DP was highest in the “Prudent” cluster, the PCA-animal protein and alcohol DP was highest in the “Non-Prudent” cluster and the PCA-convenient DP was highest in the “Convenient” cluster.

**Table 5:** Factor loadings and explained variation of dietary patterns obtained with PCA

Food groups	Dietary patterns		
	Prudent	Animal protein and alcohol	Convenient
White bread	0.02	0.01	**−0.70**
Brown bread	0.16	0.00	**0.20**
Cereals	0.13	**−0.27**	**0.40**
Pastries	−0.16	**−0.29**	0.05
Potatoes	0.00	0.12	**−0.28**
Rice pasta	−0.08	0.08	**0.21**
Fried foods	**−0.39**	0.13	−0.09
Fruits	**0.51**	−0.09	0.02
Oleaginous fruits	**0.23**	0.09	0.14
Dried fruits	**0.35**	−0.02	0.06
Soups	**0.25**	0.09	−0.11
Vegetables	**0.56**	**0.25**	0.19
Pulses	**0.27**	**0.21**	−0.06
Preserved vegetables	0.05	0.14	−0.12
Lean meat	**−0.23**	**0.38**	−0.08
Fatty meat	**−0.45**	**0.26**	0.09
Offal’s	0.15	**0.29**	−0.11
Processed smoked meat	−0.11	**0.26**	0.06
Processed meat	**−0.36**	**0.27**	0.08
Fish	**0.43**	**0.39**	0.16
Smoked and canned fish	0.17	**0.37**	**0.24**
Shellfish and mussels	0.06	**0.47**	**0.22**
Eggs	−0.05	0.12	−0.02
Ready meal	**−0.38**	0.08	**0.41**
High-fat dairy products	0.15	**−0.23**	−0.11
Low-fat dairy products	**0.26**	−0.04	**0.20**
Soya products	**0.20**	−0.11	**0.26**
Butter and low fat butter	0.01	−0.02	**−0.30**
Minarine and margarine	**−0.26**	0.04	−0.07
Olive oil	**0.35**	0.05	0.16
Oil rich in omega6	**0.30**	0.03	−0.13
Oil rich in omega3	0.15	−0.01	−0.15
Fresh cream and dressing	**−0.38**	−0.03	**0.24**
Light fresh cream and dressing	−0.03	0.05	**0.31**
Sugar and sweets	−0.11	**−0.49**	0.08
Salty biscuits	**−0.34**	0.06	**0.20**
Water	**0.24**	0.08	0.15
Coffee	−0.07	0.06	−0.17
Fruit or vegetable juice	−0.01	−0.15	**0.29**
Soft drinks	**−0.43**	−0.18	0.08
Diet soft drinks	**−0.20**	0.05	0.13
Beer	**−0.26**	**0.34**	−0.03
Wine	0.03	**0.38**	−0.07
Aperitifs and spirits	−0.15	**0.34**	−0.09
Tea	**0.36**	−0.10	−0.02
Explained variation in food groups, %	3.1%	2.1%	1.9%

Loading values superior to 0.2 or inferior to -0.2 were in bold

## Discussion

The main objective of this study was to test a method allowing the objective selection of the most appropriate model among different clustering methods and numbers of clusters in the field of “dietary pattern analysis.” The idea was to assess stability of different clustering solutions and choose the most stable solution as the most appropriate for describing the data. According to this method, three dietary patterns obtained with K-means algorithm were obtained. The “Non-Prudent” and “Convenient” patterns associated respectively with non-healthy food choices and convenient foods were both associated with a higher cardiovascular risk compared to the “Prudent” cluster characterized by healthier dietary habits and lower cardiovascular risk.

Among the clustering method considered in this article, K-means clearly showed more stable solutions regardless of the number of clusters. However, it is highly likely that other more sophisticated methods would have been more appropriate [3]. Indeed, clustering methods considered in this study were really simple and others methods with higher flexibility regarding cluster’s characteristics are more likely to identify real complex structure. In addition, although K-means was found as the most appropriate method for describing dietary patterns in adults living in the Greater region, it may not be the case with other datasets. Indeed, group structures from other populations are likely to be different. Therefore, this should be explored in additional datasets across different populations.

In the field of dietary pattern analysis, we are aware of only two studies comparing different clustering methods. Like us, Lo Siou et al. assessed stability of solutions obtained with different clustering methods and number of clusters and also showed that K-means was the most appropriate method [3]. Contrary to our results, stability decreased with the number of clusters and therefore they were not able to identify an optimal number of clusters with this method. In addition, as proposed by Lange [8], the authors also used a classifier to transfer the solution obtained on one sample to another. However, the classifier should use the same clustering method’s grouping principle. In accordance with Lange, we used the nearest-means classifier for K-means and Ward’s method and the nearest-medians classifier for K-medians. However, Lo Siou et al. used the nearest-neighbour classifier for K-means and Ward’s method. In order to assess the effect of using a not optimal classifier, we compared stability indices computed on our data with optimal classifiers and the not optimal nearest-neighbour classifier. All stability indices were lower when the not optimal nearest-neighbour classifier was used (see Additional file 2: Figure S2). Therefore, stability values computed in the paper of Lo Siou et al. may have been underestimated.

In another study, Greve et al. use an inappropriate manner for choosing the optimal number of clusters [19]. Indeed, they selected as the optimal number of cluster the number maximizing the agreement between different clustering methods. However, agreement between methods is conditioned by the capability of methods to identify cluster’s structure. Indeed, if a method is not able to distinguish clusters, it will never agree with another method even for the correct number of clusters. Therefore, although it is reassuring to have good agreement between solutions obtained with different algorithms, agreement should not be used for choosing the optimal number of clusters.

### Comparison with others studies

In accordance with the literature [2, 20], we also derived a “Prudent” dietary pattern characterized by plenty of plant foods and fish and a preference for vegetable oils and low-fat dairy products. In contrast, similar to Western DP described in others studies [2, 20], we also derived a “Non-Prudent” pattern characterized by intakes of red and processed meats, high fat content foods, refined grains, soft drinks and alcoholic beverages [21, 22]. However, contrary to most Western-DP described in the literature [2, 20], our “Non-Prudent” pattern was not associated with intakes of sweets and sugar.

Similar to some studies [4, 23, 24], we also found a cluster characterized by consumption of convenient fast foods. It showed high intakes of convenient unhealthy foods like pastries, ready meals, high-fat dairy products, fresh cream and dressings, sugar and sweets, salty biscuits, soft drinks and aperitifs and spirits. However, it was also characterized by high intakes of convenient healthy foods like cereals, preserved vegetables, smoked and canned fish, soya products, fruit or vegetable juice. Regarding nutrients, this pattern was associated with high intakes of carbohydrates, added sugar and fat.

The size of DP showed that the “Convenient” pattern was the most prevalent with 46% of the population assigned to this cluster. The “Prudent” and “Non-Prudent” patterns were adopted by 29% and 25% of the population respectively. However, striking differences were noticed across regions. Although, the “Convenient” pattern was the most adopted in all regions, the “Prudent” pattern was more frequent in Lorraine (41%) than in Luxembourg (28.7%) and Wallonia (19.1%). In sum, only a small part of the population has healthy dietary habits and this part is even smaller in Luxembourg and Wallonia. The adoption of a “Convenient” pattern may be due to the fact that people have less and less time for preparing and cooking foods and thus choose to consume prepared foods.

In line with others studies, we also found significant associations between dietary patterns and sociodemographic and lifestyle characteristics. We found that the “Convenient” pattern was more likely to be adopted by men and younger people [23]. Since the Luxembourg population is made up of more young active working people, this might explain the larger size of the “Convenient” cluster in Luxembourg compared to Wallonia and Lorraine [25]. In addition, as also shown by other studies [4], women and individuals with higher education were more likely to adopt a “Prudent” pattern. Moreover, in accordance with others studies [2, 4], we also found that people who choose unhealthy dietary habits are less likely to be engaged in healthy behaviours like doing physical activities and not smoking. It shows that the choice of a dietary pattern is in fact part of a larger pattern of lifestyle.

Concerning association with CVRF, we found that “Convenient” and “Non-Prudent” patterns were associated with higher BMI, WHR, SBP, DBP and FPG [4, 23, 26]. Moreover, the “Non-Prudent” pattern was also associated with higher HDL and LDL levels in men only. It is in accordance with others studies which also found that a cluster dominated by alcohol was directly associated with HDL [27–29]. The fact that the association was significant in men only might be explained by different level of alcohol consumption between men and women. Indeed, when clusters were described by gender, we observed that the “Non-Prudent” cluster was characterized by high intakes of alcohol in men but not in women (data not shown). Another explanation could be a different effect of diet on plasma lipids between men and women, possibly due to hormonal and sex differences in cholesterol metabolism [2, 30, 31]. Moreover, the genetic variation in lipoprotein metabolism may also have an effect [32].

### Comparison between PCA and cluster analysis

Despite clear differences in approaches and interpretation, PCA and cluster analysis gave similar results. A “Prudent” DP was identified with both methods. Indeed, a “Prudent” and “Non-Prudent” cluster with respectively high and low values on PCA-Prudent DP were found. Likewise, a convenient cluster was made of individuals with high values on PCA-convenient DP. Concerning PCA-Animal protein and alcohol pattern, we did not observe a cluster of individuals with only high intakes of meat, fish and alcohol. However, since this DP is characterized by high intakes of foods (meat and alcohol) usually consumed in a “Non-Prudent” pattern, it was significantly higher in the “Non-Prudent” cluster. Those results are in line with others studies, which also found differences in mean PCA-DP across clusters [33–35].

Although results between both methods were similar, they describe diet in different ways. Indeed, PCA aims to determine DP explaining variation in a set of food groups whereas cluster analysis aims to identify groups of people with different food intakes. Moreover, the format of DP is also different. An individual’s dietary pattern is described through his/her membership to a group in cluster analysis whereas in PCA-DP the subject is described with his/her scores on all computed DP. Therefore, the choice of a method depends on both the desired format of the outcome but also hypothesis and aims of the study. Advantages of PCA are that it may be easier to perform as it requires less subjective researchers’ decisions. However, findings from cluster analysis are easier to interpret because an individual is assigned to one cluster only whereas PCA-DP do not refer to identifiable groups within the population, and hence do not give an indication of the prevalence of a particular type of diet [35]. On the other hand, continuous factors determined by PCA may be advantageous when relationships between DP and others variables are assessed since a gradient is formed between individuals with low, medium or high values on factors. Moreover, they do not require the use of a reference category [26]. As other authors have suggested, unless the choice of one method is justified, it is advisable to use both factor and cluster analysis in order have complementary insights [36].

### Strength and limitations

The main strength of this study was the use of an objective procedure to select the most appropriate clustering method and number of clusters. Compared to other internal validity indices, the stability measure has the advantage to be model free and not being optimized by any clustering method. Moreover, comparison of cluster solution and PCA-derived factors were also made. Further, this study used a recent and homogeneous design of data collection including three large randomly selected samples from three neighbour regions. Shortcomings of this study were that considered clustering methods were all heuristic-based and make basic assumptions on group structure. The reason is that since the main objective of this study was to test the objective procedure, we decided to limit its application to traditional clustering methods used in the field of dietary pattern analysis [2]. Therefore, we will also consider more sophisticated methods in the future. In addition, although the method allows distinguishing between stable and spurious clustering solutions, stability is not the only aspect of a good solution. Indeed, a stable clustering solution may still be meaningless if it does not discriminate useful subset of the overall data [37]. However, unstable solutions should not be interpreted and thus stability is an indispensable requirement [37]. For this reason, the interpretation and criticism of the clustering solution by the researcher and comparison with results obtained with PCA are still important. In addition, many others subjective decisions have still been made that are likely to influence the final solution, namely the pooling of different food items into specific food groups, the quantification of the input variables, the adjustment for total energy intake and the method of standardization. However, the robustness of the chosen solution and the consistency of the results with PCA-DP gave confidence in our results. Other limitations are the cross-sectional design of the study and the probable measurement error linked with the FFQ. Finally, although we identified dietary pattern associated with disease risk, we still do not know if this effect comes from certain component only or is the product of the addition or interaction of several food groups.

## Conclusion

In summary, we used an objective methodology based on the stability of clustering solutions allowing selection of the most appropriate clustering method and number of cluster for describing dietary patterns in a population. Three main dietary patterns were identified in the Greater region. A “Convenient” and a “Non-Prudent” pattern associated with a higher cardiovascular risk and a “Prudent” pattern associated with a decreased cardiovascular risk. Those results flag the need for targeted public health initiatives promoting the benefit of a prudent dietary pattern and other healthy behaviours to relevant subgroups like men, young and less educated people, at interregional level.

## Additional files

Additional file 1: Figure S1. Flowchart of participants who met inclusion criteria (PNG 46 kb)
Additional file 2: Figure S2. Distribution of stability indexes across clustering methods and number of clusters by type of classifier (PNG 138 kb)

## Abbreviations

ARI: Adjusted rand index
BMI: Body mass index
CA: Cluster analysis
CVRF: Cardiovascular risk factors
DBP: Diastolic blood pressure
DP: Dietary patterns
FFQ: Food frequency questionnaire
FPG: Fasting plasma glucose
HbA1c: Glycated hemoglobin
HDL: plasma high density cholesterol
LDL: Plasma low density cholesterol
MUFA: Monounsaturated fat
OR: Odds-ratio
PCA: Principal component analysis
PUFA: Polyunsaturated fat
RRR: Reduced rank regression
SBP: Systolic blood pressure
SD: Standard deviation
SFA: Saturated fat
TC: Plasma total cholesterol
TG: Triglycerides
WHR: Waist to hip ratio
