# Supplementary figures and images for: Stability-based validation of dietary patterns obtained by cluster analysis
Source: Nutr J. 2017 Jan 14;16:4. doi: 10.1186/s12937-017-0226-9 (PMC5237531; doi:10.1186/s12937-017-0226-9)

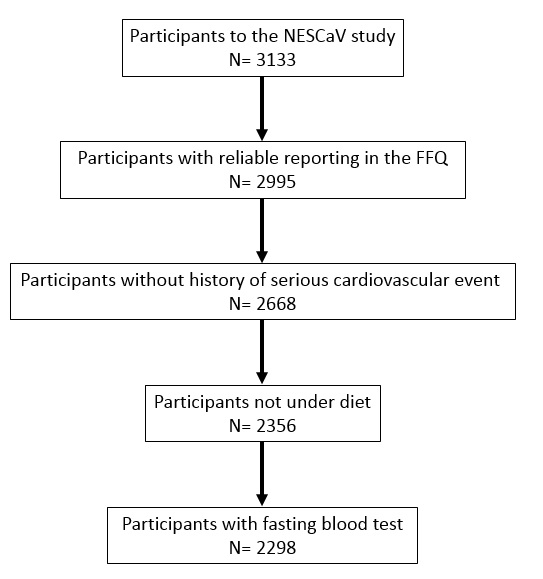

Supplement: Additional file 1: Figure S1. — Flowchart of participants who met inclusion criteria (PNG 46 kb) [file 12937_2017_226_MOESM1_ESM.png]

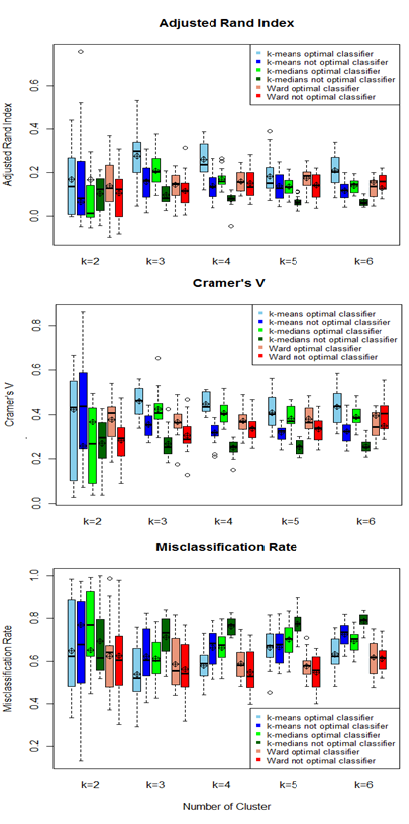

Supplement: Additional file 2: Figure S2. — Distribution of stability indexes across clustering methods and number of clusters by type of classifier (PNG 138 kb) [file 12937_2017_226_MOESM2_ESM.png]
